# “Semen-oma”: Sequestered Semen Collection From Urethral Stricture and Bladder Neck Closure in Exstrophy Reconstruction

**DOI:** 10.7759/cureus.70162

**Published:** 2024-09-25

**Authors:** Hannah Baker, Mark Rich, Javier Miller, Lucas R Wiegand

**Affiliations:** 1 Urology Department at Orlando Health, Florida International University, Herbert Wertheim College of Medicine, Miami, USA; 2 Pediatric Urology, Orlando Health Arnold Palmer Hospital for Children, Orlando, USA; 3 Urology, Orlando Regional Medical Center, Orlando, USA

**Keywords:** bladder exstrophy-epispadias complex, bladder neck obstruction, ejaculatory duct obstruction, semen sequestration, urethral stricture

## Abstract

Ejaculatory dysfunction in adult males with bladder exstrophy-epispadias complex can occur from associated genitourinary anomalies, surgical iatrogenic scarring, infection, obstruction, and neurologic and functional causes. This case presents a 30-year-old male patient with a history of bladder exstrophy reconstruction (bladder neck closure and appendicovesicostomy) who presented with a nine-year history of intermittent perineal scrotal pain, swelling, and intermittent urethral discharge. He presented with a tender palpable perineal mass (“semen-oma”). Examination under anesthesia and endoscopy of the urethra demonstrated a distal anterior penile and proximal urethral stricture at the level of the bladder neck level from previous surgical exstrophy reconstructive procedures. Aspiration of the mass revealed a thick viscous fluid suggestive of semen. Intraoperative endoscopic evaluation and radiographic imaging revealed a distal penile urethra stricture and proximal bladder neck closure from previous genitourinary reconstruction with sequestration of semen in the ectatic urethra with reflux into the seminal vesicle. A proximal ventral penile urethrostomy was performed to allow for permanent drainage of the sequestered semen collection and future antegrade ejaculation. The postoperative course was uneventful. This case highlights the potential for sexual ejaculatory dysfunction and the need for long-term and transitional urologic care in patients with a history of complex exstrophy reconstruction.

## Introduction

Bladder exstrophy-epispadias complex (BEEC) includes a spectrum of congenital genitourinary anomalies that challenge pediatric and adult reconstructive urologists. With improved medical care and surgical reconstruction, more patients with complex urologic conditions, including bladder exstrophy, are living into adulthood and require a transition from pediatric to adult urologic care [1]. It is common for males born with bladder exstrophy/epispadias to have genitourinary issues throughout pediatric and adult life that require additional urologic procedures to reconstruct and restore the anatomy and function of the genitourinary system [2]. Encountering different types of surgical reconstructions is common when managing these patients. Sexual ejaculatory dysfunction in adult males with BEEC may occur from a variety of congenital anomalies associated with exstrophy, including deficiency of the urethra and bulbospongiosus muscles, prostate, ejaculatory ducts, bladder neck, kidneys, ureters, iatrogenic surgical procedures, infectious, neurologic innervation, and functional causes [3]. In this case, we describe the presentation and diagnostic and surgical management of an adult male presenting with iatrogenic ejaculatory dysfunction secondary to previous complex genitourinary reconstruction performed for the repair of bladder exstrophy.

## Case presentation

A 30-year-old male presented with a nine-year history of intermittent groin and perineal pain with perineal and left scrotal swelling relieved with the spontaneous discharge of viscous semen-like emission from the urethra. The patient had a complex history of ambiguous genitalia with bladder exstrophy and imperforate anus. Multiple surgical and urologic procedures were performed throughout his life, including posterior sagittal anorectoplasty, exstrophy repair, left nephrectomy for left renal dysplasia-associated ureteral ectopia to left seminal vesicle, and repair of ambiguous genitalia. Additional urologic surgery performed at age nine for urine incontinence was based on a small neurogenic noncompliant bladder, patulous bladder neck, entailing ileal bladder augmentation cystoplasty, and closure of the bladder neck with the creation of a continent catheterizable appendicovesicostomy for bladder clean intermittent catheterization (CIC).

The patient was dry and performed intermittent catheterization every four to six hours and bladder irrigation. At age 15 years, he presented with a bladder perforation due to poor bladder management with catheterization compliance. At age 18 years, he developed recurrent urinary tract infection (UTI) with bladder stones and underwent flexible ureteroscopy through an appendicovesicostomy and laser lithotripsy. He subsequently was compliant with bladder management, CIC, and bladder irrigation and remained stable from a urologic perspective until the transition to adult care at age 21.

**Figure 1:**
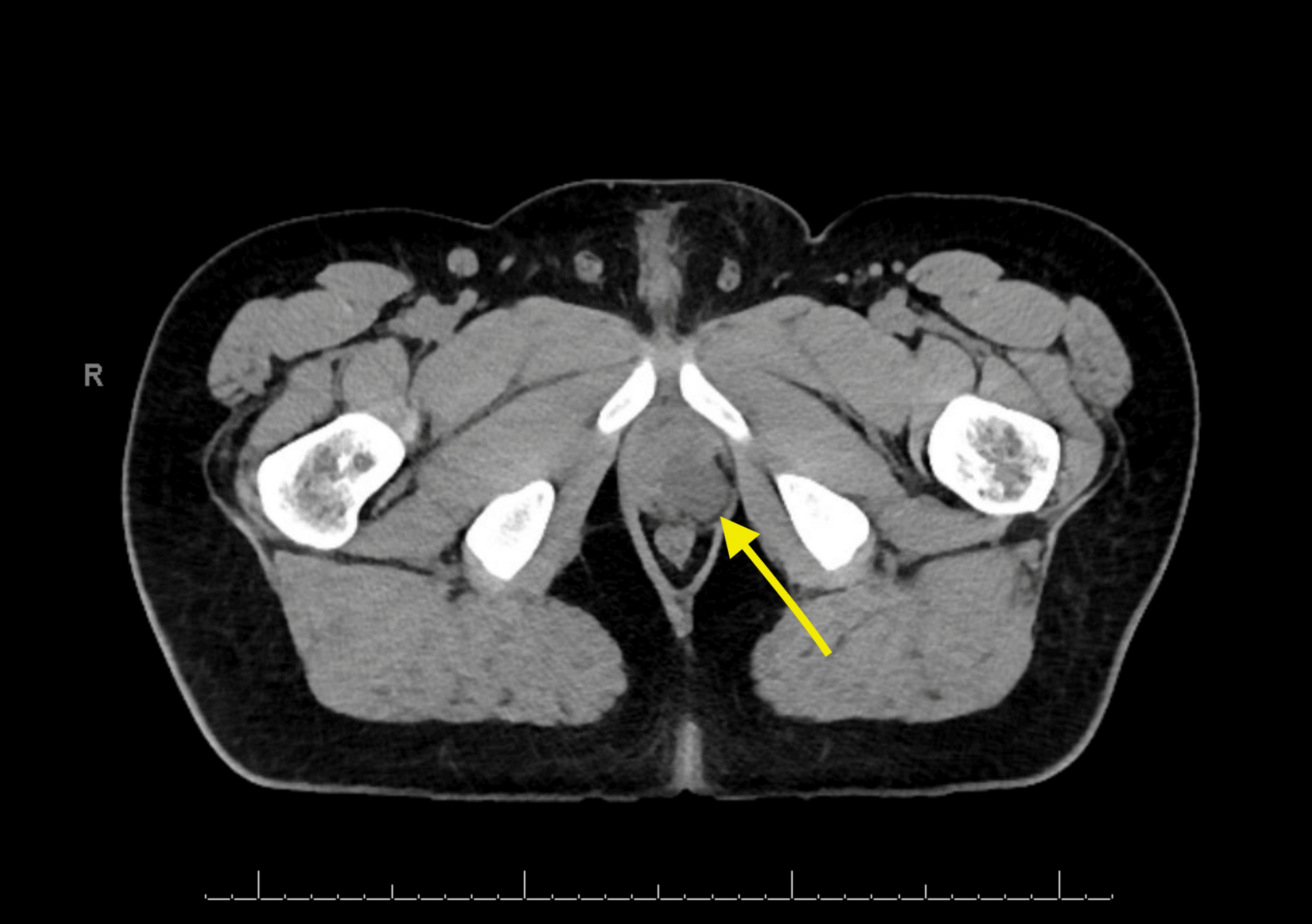
CT of the pelvis with the yellow arrow demonstrating dilated seminal vesicle.

At 21 years at his ultimate transitional urologic visit, he denied UTI, stones, or incontinence. He had a sexual history of normal erections and orgasm without ejaculation and reported nocturnal emission from the urethra. He presented with a new complaint of intermittent left scrotal and perineal pain and swelling. Ultrasound of the scrotum and CT and MRI of the pelvis demonstrated bilateral pelvic sidewall lobulated cystic lesions consistent with dilated seminal vesicles extending to the left base of the penis (Figure 1). The patient had a resolution of pain and swelling with spontaneous emission from the urethra.

**Figure 2:**
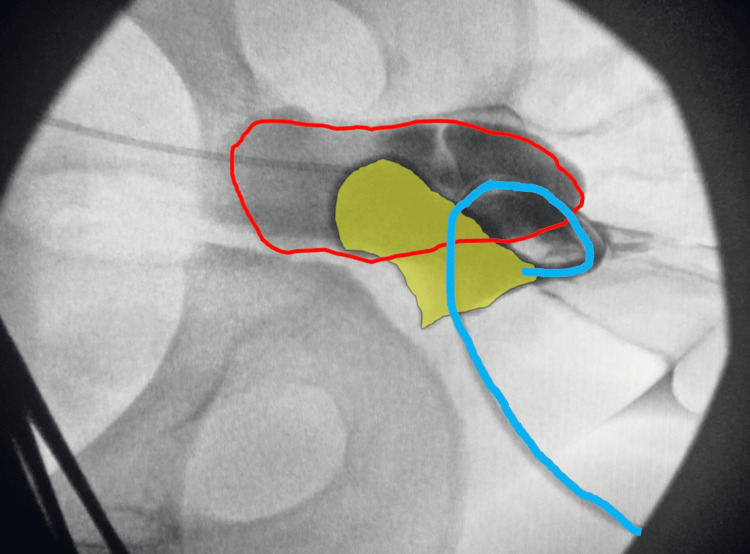
Urethrogram showing a dilated prostatic urethra (shaded yellow) and reflux into the seminal vesicle (red outline). The blue line is the catheter and urethra distally.

**Figure 3:**
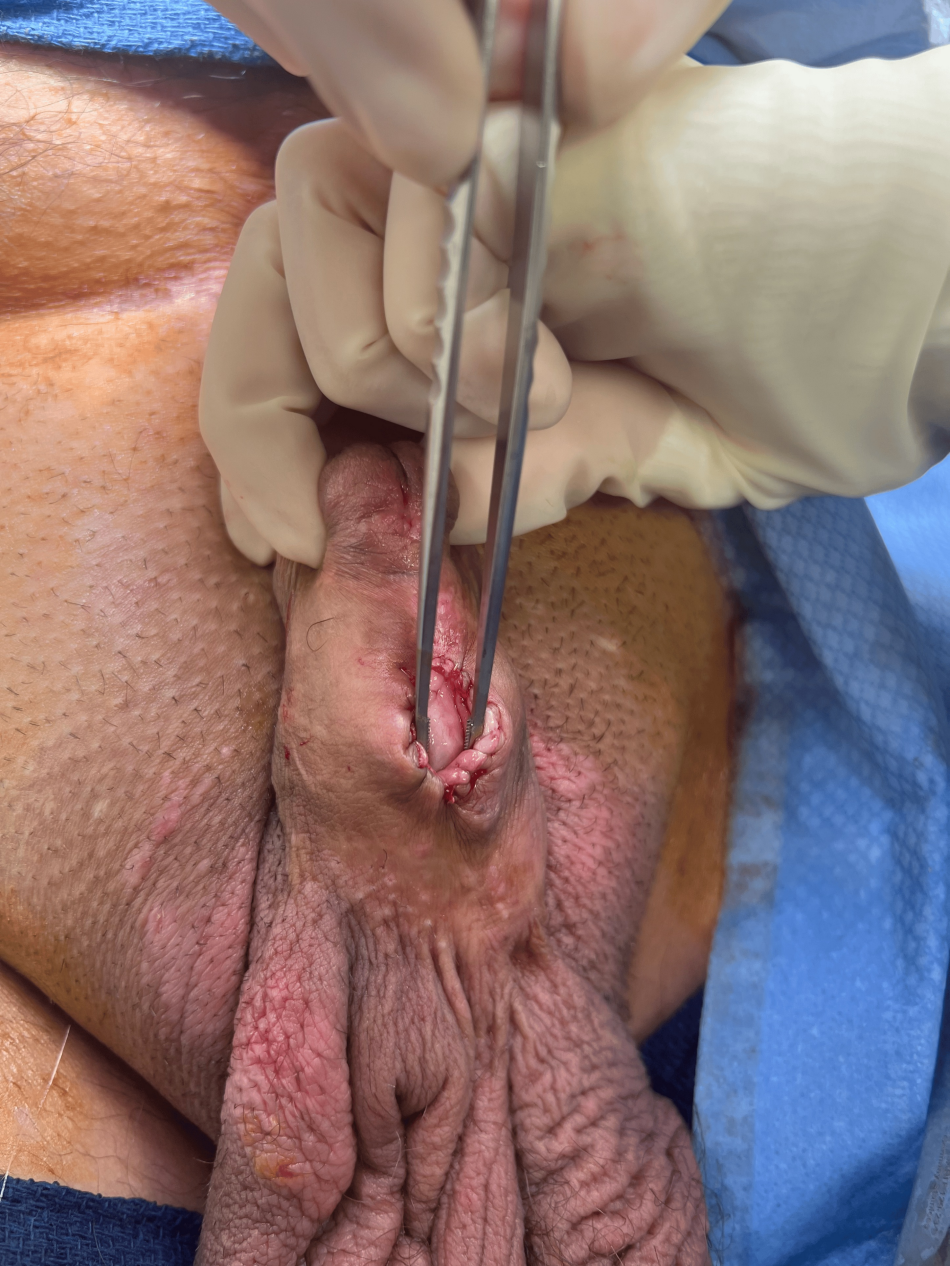
Penile urethrostomy to allow passage of semen using forceps in urethrostomy.

Ultimately, the patient developed anejaculation but noticed increased swelling of the urethra/penile mass with each orgasm. The patient reported self-drainage of fluid from the affected area by needle aspiration every few months. Ultimately, he sought consultation with adult urology and was evaluated under anesthesia for the diagnosis and treatment of sequestered semen. The procedure was performed in the lithotomy position. Diagnostic urethroscopy demonstrated a distal penile stricture at the level of the fossa navicularis. Aspiration was performed with an 18-gauge needle of thick seminal fluid from the urethra and a guidewire was inserted through the needle into the urethra under fluoroscopic guidance. A 5-French open-end catheter was inserted over the wire and a retrograde urethrogram demonstrated a dilated urethra extending proximally to the prostate with reflux into the left seminal vesicle (Figure 2). A 16-French catheter was inserted into the bulbar urethra draining 200 mL of cloudy milky seminal fluid. Proximal urethroscopy was performed confirming the findings on the urethrogram (Video 1). A proximal ventral urethrostomy (Figure 3) was created to allow drainage of the sequestered semen from the urethra, prostate, dilated genital ejaculatory ducts, and seminal vesicle.

**Video 1:** Urethroscopy showing closed bladder neck and dilated prostatic, membranous, and bulbar urethra.

## Discussion

Bladder exstrophy includes a rare complex spectrum of congenital genitourinary anomalies involving the abdominal wall, bladder and urinary tract, genitalia, bony pelvis, spine, and anus with a prevalence of around 3/100,000 live births with a 2:1 male predominance [1].

Surgical treatment of bladder exstrophy not only involves bladder closure but also includes procedures such as genital epispadias repair, bladder neck reconstruction, bladder augmentation, and ureteric reimplantation. Various surgical procedures have been described for the reconstruction of bladder exstrophy. They fall into the following two main categories: reconstructive procedures and diversion procedures [4]. In this case, enterocystoplasty was performed for a patient with low bladder capacity and poor compliance. Morbidity and complications can arise from multiple surgical reconstructions, including bowel obstruction, bladder perforation, and stones, as well as future malignant transformation and medical issues from recurrent UTI, gastrointestinal malabsorption, and metabolic complications of urinary intestinal diversion and reconstruction [5,6].

Adult men with exstrophy are capable of ejaculation and orgasm, although semen parameters can be affected by ejaculatory dysfunction. Reasons for ejaculatory dysfunction include surgical scarring and iatrogenic injury to the vas deferens, ejaculatory ducts, verumontanum, prostate, urethra and bladder neck, recurrent UTI and epididymo-orchitis, retrograde ejaculation, and deficiency of urethral bulbospongiosus muscle [7].

## Conclusions

To our knowledge, this rare and unique case is the first report of the creation of a ventral urethrotomy for the treatment of semen sequestration, “semen-oma,” and highlights the potential of lifelong urologic and sexual issues in adult men with complex congenital anomalies and importance for transition to adult urologic care.
